# Supplementary material for: Discrimination of Lung Cancer and Benign Lung Diseases Using BALF Exosome DNA Methylation Profile
Source: Cancers (Basel). 2024 Aug 5;16(15):2765. doi: 10.3390/cancers16152765 (PMC11311347; doi:10.3390/cancers16152765)
Supplement: Supplementary file 1 [file cancers-16-02765-s001.zip › cancers-3069229-supplementary.pdf]

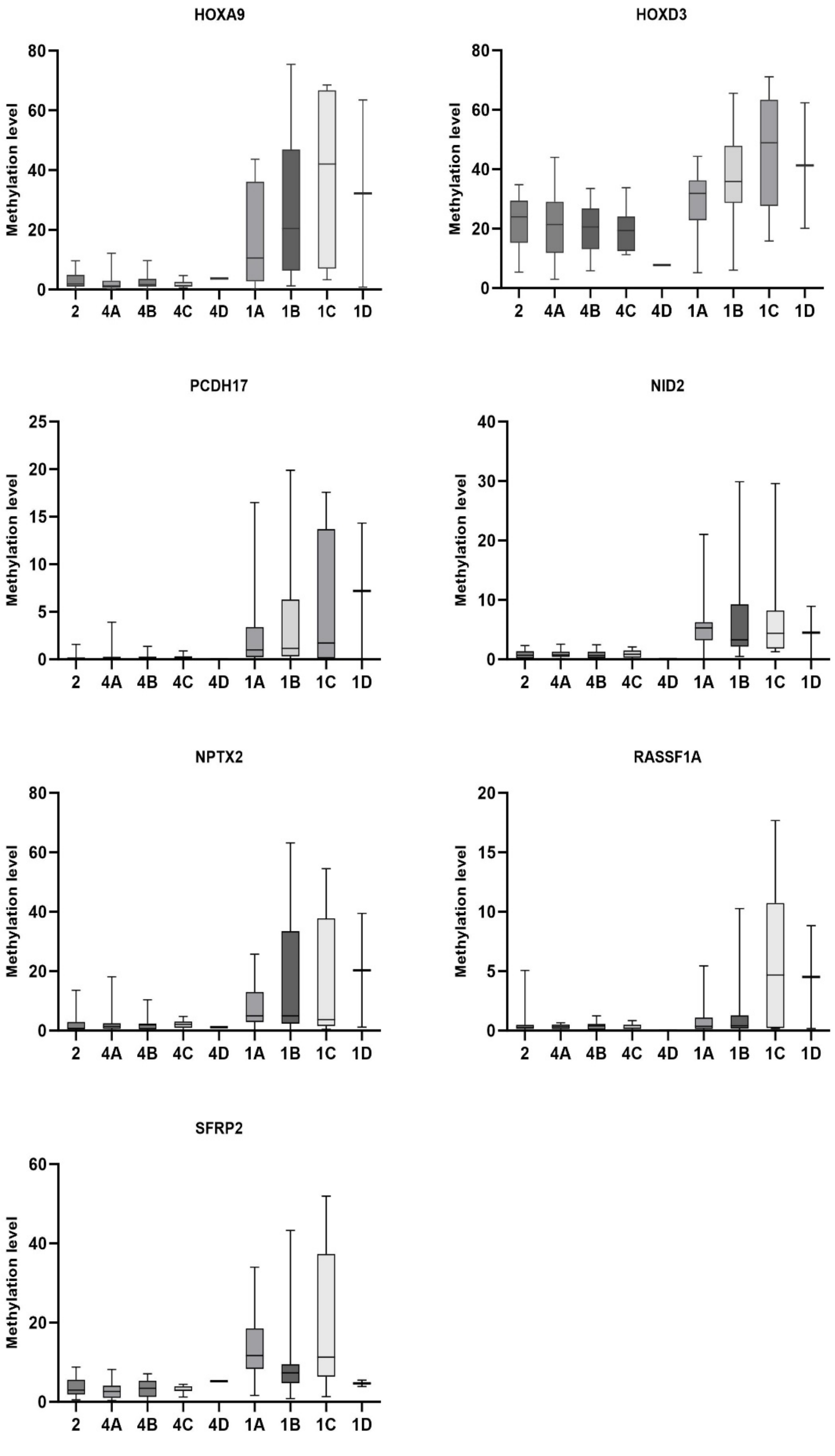

Supplementary Figure S1. The mean PMRs of each marker among non-cancer and cancer subtypes. 2: Nodule; 4A: Pneumonia; 4B: Tuberculosis; 4C: Chronic Obstructive Pulmonary Disease; 4D: idiopathic pulmonary fibrosis; 1A: Adenocarcinoma; 1B: SqCC; 1C: SCLC; 1D: LCLC

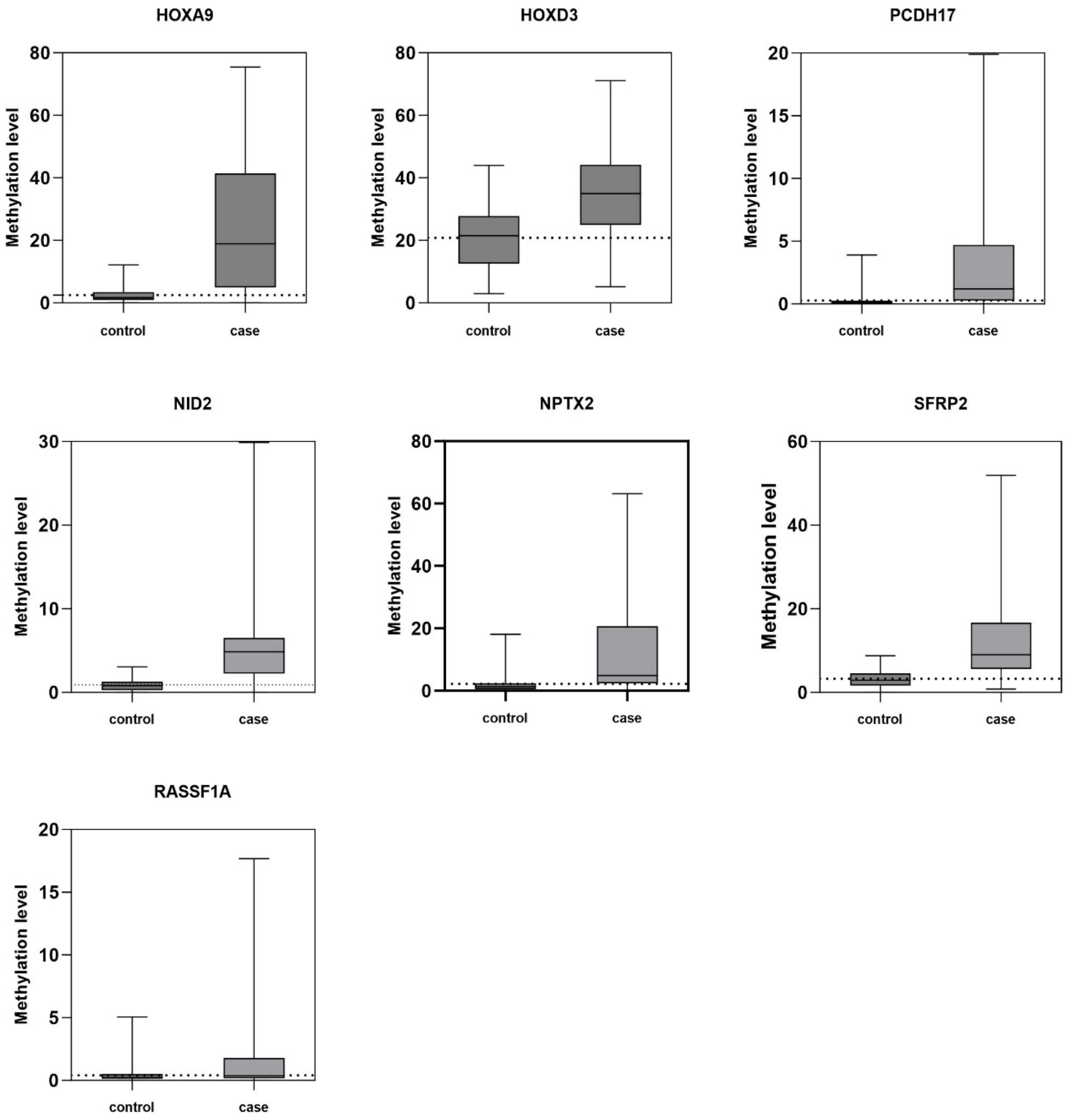

Supplementary Figure S2. The mean PMRs of each marker in non-cancer and cancer subtypes. Control: Mean of the PMRs of all non-cancer samples; Case: Man of the PMRs of all cancer samples.

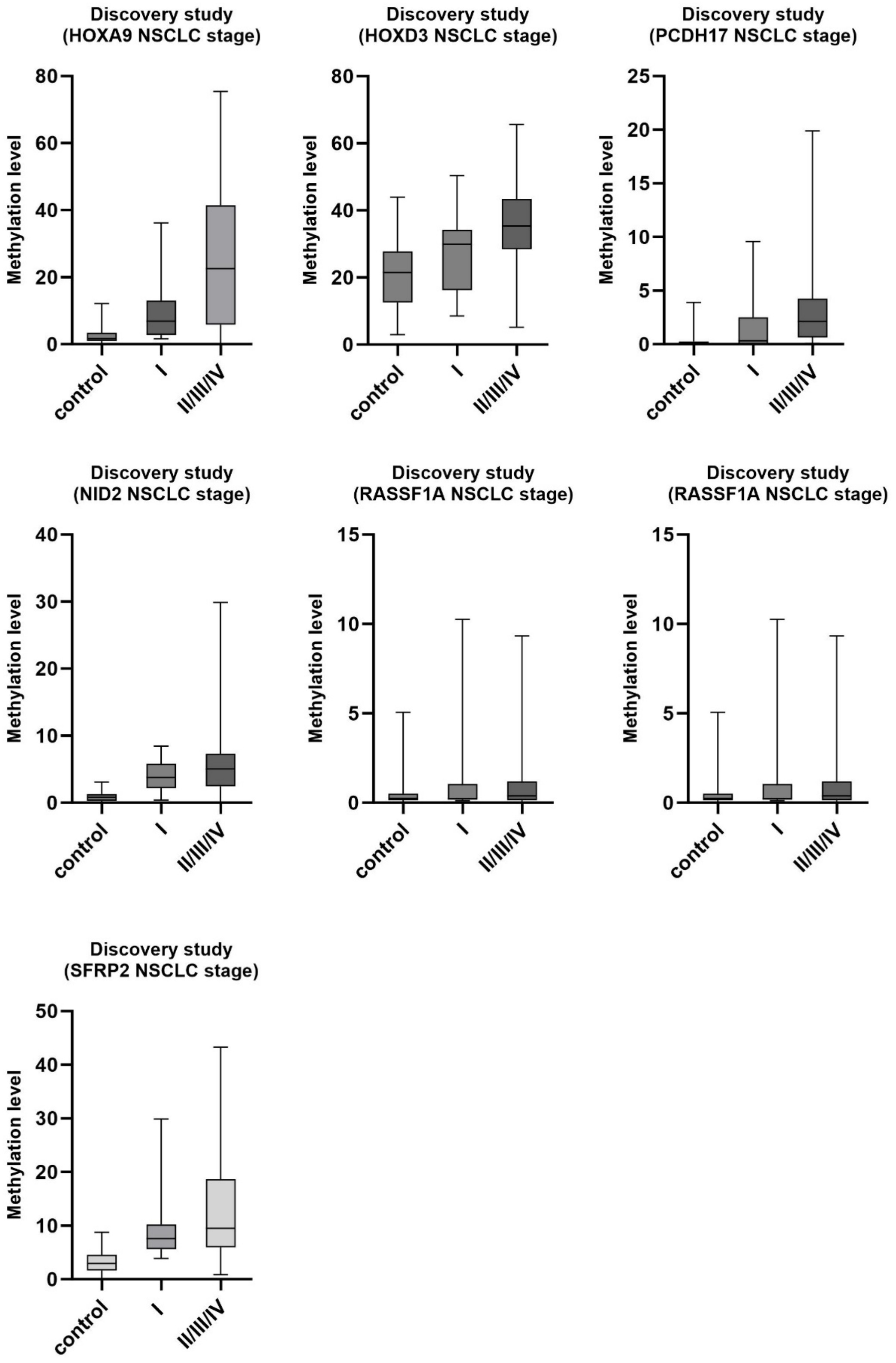

Supplementary Figure S3. The mean PMRs of each marker in non-cancer and NSLC progression. Control: Mean PMRs of all non-cancer samples; I: Mean PMRs of NSCLC stage I samples; II/III/IV: Mean PMRs of NSCLC advanced stage samples.
